# Morpho-elasticity of inflammatory fibrosis: the case of capsular contracture

**DOI:** 10.1098/rsif.2015.0343

**Published:** 2015-10-06

**Authors:** Martine Ben Amar, Min Wu, Miguel Trejo, Michael Atlan

**Affiliations:** 1 Laboratoire de Physique Statistique, Ecole Normale Supérieure, UPMC Univ Paris 06, Université Paris Diderot, CNRS, 24 rue Lhomond, 75005 Paris, France; 2 Institut Universitaire de Cancérologie, Faculté de médecine, Université Pierre et Marie Curie-Paris 6, 91 Bd de l'Hôpital, 75013 Paris, France; 3 Laboratoire de Physique et Mécanique des Milieux Hétérogènes (PMMH), UMR CNRS 7636; PSL - ESPCI, 10 rue Vauquelin, 75005 Paris, France; 4 Hopital Tenon APHP, Université Pierre et Marie Curie-Paris 6, 3 rue de la Chine, 75020 Paris, France

**Keywords:** fibrosis, pathology, soft tissues, nonlinear elasticity, inflammation, patient biopsy

## Abstract

Inflammatory fibrosis is a wound-healing reaction of the immune system in mammals against aggression. After a signalling cascade, fibroblasts and potentially myofibroblasts make a stiff collagenous tissue inside the body that modifies the original healthy tissue. We focus here on the implant-induced fibrosis that aims to encapsulate the implant with a typical fibrous tissue called the capsule. Focusing on breast capsules, we aim to understand the mechanical properties of these tissues, to test the validity of fibre models that have been established in other contexts such as arteries. For this purpose, we perform force–extension experiments and show that mechanical constitutive laws of these tissues are especially difficult to derive, because models are sensitive to fibre orientation and dispersion, independently of the variation between individuals. In addition, fibre breakdown, and possibly remodelling, occur during the extension experiments. However, the high stiffness of the capsular tissue, compared with the healthy tissue, added to the fact that an inflammatory process has no reason to cease, is at the origin of large compressive stresses *in vivo*, which explains the pain and unaesthetic deformity. We evaluate the stresses responsible for the pain and the buckling instability, which have no reason to stop if the inflammation persists.

## 1. Introduction

Fibrosis, the formation of excess fibrous connective tissue, seems to be a common mechanism in organisms to find for protection, foreign body reaction and survival. Put on a solid substrate, a drop-containing bacteria (*Bacillus subtilis*) [1] extends by constructing a fibrous gel called biofilm. On a liquid substrate, it makes a solid thin plate the elasticity of which has been shown to be anisotropic both in tension and compression [2]. The fibrous matrix adheres strongly and organizes the cell division. For humans, fibrosis occurs as a reaction of the immune system against aggression: wounds [3,4], solid tumour growth [5,6], implants [7] and also severe obesity. When it becomes excessive, it induces pathological complications, unaesthetic in the best cases, painful and life threatening in the worse cases. Moreover, the fibrotic tissue, which is difficult to eliminate physically, limits the transport of drugs, and the preferred solution remains surgery.

The introduction of a foreign body in mammals causes an immediate wound-healing response with a complex signalling cascade [8]. In the case of breast implants, the final and long-term result is encapsulation of the implant by an inflammatory collagenous tissue called the capsule. Helpful not only to fix it in the breast, but also to prevent infection and trauma, the capsular tissue may stiffen and extend, becoming extremely painful and unaesthetic only few months post-implantation. Several causes have been investigated such as bacterial infection [9,10], previous breast and chest irradiation, and the structure and surface texture of the implant. However, few histological studies have been published regarding fibrosis [7] and clearly, such information is essential to fully understand the process.

The biomechanics of growing soft tissues is a recent domain of study which covers plant morphogenesis [11], embryogenesis [12], organ development [13] and pathologies [5,6]. Many studies have been undertaken in the last few years to understand the growth and form of biological objects in the spirit of Thompson [14] by considering the shape instabilities of growing simple geometric objects: spheres for tumours, cylinders for arteries, plates for skin. These studies oversimplify the structure of the tissues, being simply represented by ad hoc constitutive laws and treated in the context of finite elasticity with volumetric growth [12,15]. Indeed, organ tissues have a microscopic structure: epithelia mostly comprise well-ranked layers of cells, whereas connective tissues include cells, fibres and vessels in addition to fluids. Constitutive laws represent an average behaviour that is supposed to take into account the fine microscopic structure. Their validity is tested by the treatment of simplified situations.

The present study is a biophysical and biomechanical investigation of the growth of collagenous tissue owing to inflammation. Our basic system of study will be the capsular tissue around breast implants [7] and its excessive contracture, but will not be limited to it. When it occurs, in the worse cases, the implant is crumpled or fractured, with visible deformity of the breast and pain. The deformations of the breast and implant will be explained by a buckling instability inducing a shape bifurcation owing to constrained volumetric growth [15,16]. To reach this objective, however, requires a good representation of the elasticity of such tissue. We will take into account the elasticity of the tissue as derived from uniaxial traction experiments to propose the best modelling of capsular tissue. However, when constrained by the implant, which plays the role of an imposed boundary, volumetric growth will generate compressive stress that increases with the thickness of the current layer. Even in the simplest case, such as homogeneous volumetric growth of a neo-Hookean elastic sample [16], the tissue and the implant will buckle owing to the compressive stresses induced by growth. Because fibrotic tissues are more complex, we expect stronger deformations, which we aim to quantify.

The stresses may have a double origin: passive or/and active. Passive elasticity for living tissues, or dead elasticity [14], which treats living matter as a soft inert material. When the structure evolves over a very long period of time compared with the short timescale of elasticity (of the order of a second), the global shape of the sample retains a minimum of the elastic energy. Active elasticity [17] infers that the sample contains specific cells acting like small compressive motors or point stress sources. Myofibroblasts, having originated from the immune system, may play this role, contributing to the pathology. In case of adult wound-healing as an example [3,18], they are responsible of the final closure of wounds. A good model may help to distinguish between both contributions although we suspect, without precise observations, that these active cells do not exist at the early stages of the disease process. Even remaining at the level of passive elasticity, an appropriate constitutive law is required, and one part of this work is devoted to determining this law via traction tests. To the best of our knowledge, we have performed these tests for the first time, on post-surgical tissues obtained post-surgery. Although preliminary, the present study is sufficient to establish a good model of capsule tissue deformation as a function of its extension.

The paper is organized as follows. In §2, we explain the surgery and the experimental tests. In §3, we introduce the model of extension of an incompressible soft-matter cuboid compared with standard predictions for the ground matrix. In §4, we introduce the existence of the cross-linked fibre network in the hyperelastic models. We select two models, and we introduce fibre dispersion for the orientation and filament breakages. In §5, we calculate the stresses that appear in the capsule as the fibrosis occurs using our measurements. Finally, in §6, we offer some concluding remarks.

## 2. The problem, material and methods

The peri-prosthetic capsule is a normal physiological response to a foreign object introduced in human body. Soon after surgery, the implanted prosthesis becomes surrounded by an immature tissue made of fibrin mostly and phagocytes. In approximately four weeks collagen and inflammation lead to the formation of a mature capsule. Capsular contracture is the excessive fibrosis around the implant that leads to a high re-operation rate. According to the International Society of Aesthetic and Plastic Surgery, for aesthetic breast augmentation, complication rates are 1% per year, and after 10 years, the rate for capsular contracture exceeds 10% but is approximately 25%, in the case of reconstructive surgery after breast cancer treatment. According to Moyer *et al.* [7], the organization of collagen fibres around the implant evolves from loose organization to a well-spaced thick collagen fibre network when the severity increases (measured by a clinical index or grade) from Baker grade I up to IV. In addition, cells, such as mast cells, present in less dense breast capsules appear to be absent from more advanced fibrosis. In this case, fibroblasts organize themselves parallel to the fibres or in a spiral fashion. The collagen network is cross-linked and more or less parallel to the implant surface. In more advanced stages, muscle-like cells are recruited, contributing to a higher state of stress. A more quantitative study [19] has been published recently, covering capular contracture cases of all grades. It confirms the increase in the density of the collagen fibres with grade, the alignment parallel to the surface device (loosely oriented for low grade, well oriented for contracted capsules) and the presence of myofibroblasts for grade IV capsules [19]. For irradiated patients, the histological damage and changes of the breast skin [20] increase the probability of high-grade capsular contracture.

### 2.1. Specimen preparation

Nine biopsies were taken from patients: five with Baker III and IV capsular contracture, after implant-based reconstruction, and four cases concerned breast augmentation for aesthetic purposes (grade I). Two patients of the first category have previously received irradiation treatment [20,21] after partial mastectomy. Samples were harvested from surgical specimens of capsulectomy (anterior and posterior). Each sample was cut from the anterior part of the surgical specimen. All patients received the same brand of implant, the same grade of silicone gel and shell texture (ALLERGAN 410, Anatomical textured implant, texture: biocell). The dimensions of the samples were identical, 1 cm in width, 3 cm in length. The thickness increased with capsular contraction severity. The specimens were treated in less than 2 days (stored in a sterile saline solution) for tension tests. Once they are excised, the stress owing to *in vivo* confined growth is eliminated, but solid stresses or residual stresses may remain in the absence of loading. These stresses are the active part we discuss in the Introduction or come from plastic reorganization of the collagen or remodelling during contracture. The best way to identify such stresses is to carefully cut cuboids of the tissue and observe the shape as time goes on, make an incision and study the aperture, in other words to play with simple shapes immediately post-surgery, then examine them after few hours.

The deformation of the sample if it occurs after a cut indicates the existence of residual stresses as shown by Fung [22], more recently for desmoplastic solid tumours in references [5,6] for example. An opening indicates a tensile stress and a cusp-like opening indicates a contractile state followed by a tensile one. With such techniques, Stylianopoulos *et al.* [5] were able to evaluate the stresses stored during tumour growth of a few kilopascals with a map of their orientation inside the tumour. Further evidence for the presence or absence of residual stresses may be given simply by the behaviour of the uniaxial signal force versus stretch for low loadings. For the set of samples covered by this study, no obvious evidence was made of possible residual stresses or of a high density of active cells.

### 2.2. Experimental set-up and protocol

**Figure 1.**
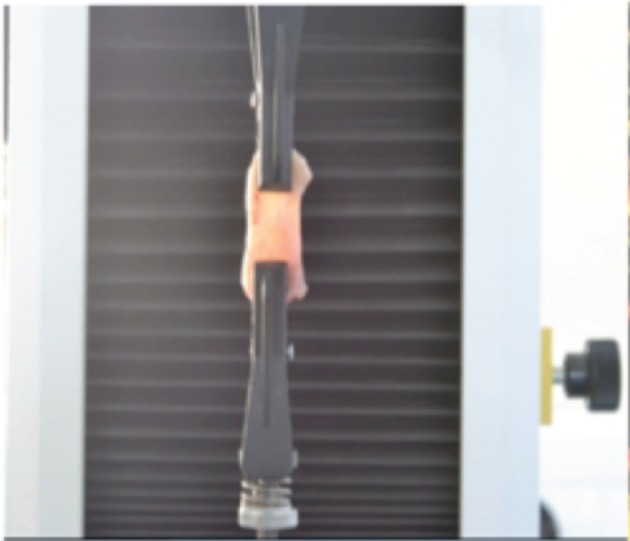
A schematic example of capsular tissue attached to the traction apparatus. Traction tests are performed at constant velocity along in the vertical *X*-direction.

**Figure 2.**
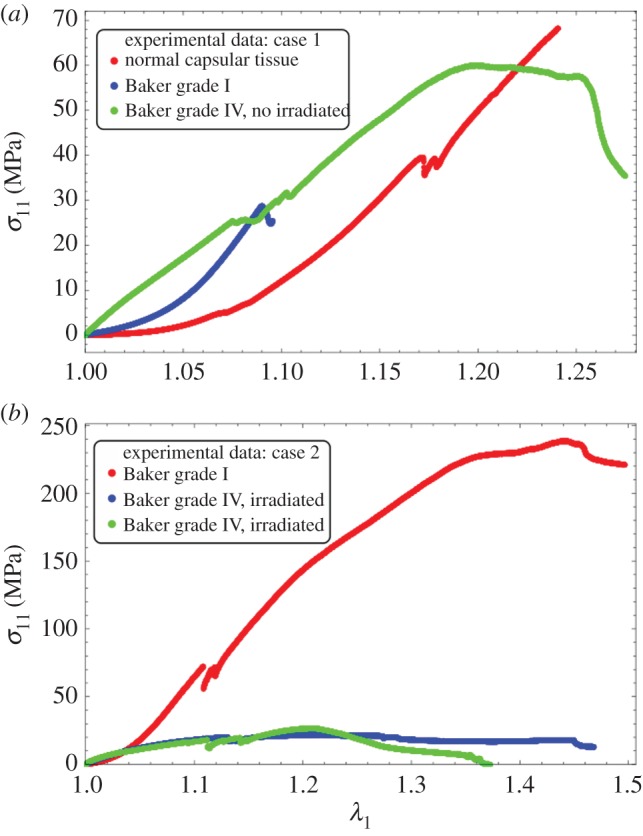
Experimental results: (*a*) experimental stress–stretch relation for different capsular tissues. The orientation of the fibres is unknown. The curves correspond to three different degrees of severity of fibrosis. The surgery corresponded to a case of breast augmentation for aesthetic purposes. None of the patients had undergone previous irradiation treatment. (*b*) Experimental stress–stretch relation for different capsular tissues. The orientation of the fibres is also unknown. The surgery corresponded to a case of an implant-based reconstruction post-cancer. The patient in this case had previously undergone irradiation treatment.

Uniaxial tension tests of the aforementioned capsular tissues were performed in a testing set-up shown in figure 1. The samples were attached to the machine using pneumatic grips prohibiting the sliding of the tissue that may occur during the tensile test, 1 kPa being the maximal compression exerted by the grips. The testing apparatus was an INSTRON (Instron 3343 1 kN single column testing systems) with a load cell that allowed measurement of vertical force in the range from 10^−3^ to 10^3^ N (with a precision 1 µN) for loading velocities between 0.005 and 500 mm min^−1^ (precision of 1 µm s^−1^). The vertical displacement (along X) is measured directly by the traction machine. Some experiments were documented by automatically taking CCD camera images. The images were used to ensure that during the tensile test, samples contract in the middle section of the sample, whereas the upper or lower faces of the sample were maintained in the pneumatic grips. The tests were performed at room temperature (*T*_e_ ≈ 21°C). During sample preparation, it was verified that no residual stresses or internal cell activity were present in the tissues by cutting the tissues and observing their evolution in time. Tissues were cut in rectangular ribbons of different sizes in the following range: initial length: 2.0–5.0 cm, initial width: 0.5–2.0 cm, initial thickness: 0.8–2.0 mm. These rectangular tissue samples were axially extended between both pneumatic grips at a fixed velocity of 1 mm min^−1^. Only one loading cycle was made with each sample. One of the practical difficulties in performing tension experiments was placing samples in the testing machine in reliable and repeatable way. At the end of the procedure, no signs of dehydration were observed. At first, grips move at zero force, zero extension, then the force increases indicating the start of the loading and fixing the correct initial size of the sample. The tests were continued until the failure of the specimen. Typical results of the traction experiments performed in the capsular tissues are shown in figure 2*a,b*, which we will compare with an equivalent experiment for fabrics [23].

## 3. Determination of the Constitutive Law

### 3.1. The space of configurations and the stress calculation

We focus here on a cuboid submitted to uniaxial tension along the *X*-axis. We assume that the sample has a length *X*_0_ before stretching larger than the width given initially by *Y*_0_ (in the *Y*-direction) and a thickness *Z*_0_ (in the *Z*-direction). The tissue extends in the *X*-direction, keeping its cuboid shape. This assumption is rather tenuous, but has been checked carefully in our experiment. The surgical cuts are parallel to the implant and correspond to the *X*, *Y* plane. We look for the simplest solutions, taking advantage of the small values of *Y*_0_/*X*_0_ and *Z*_0_/*X*_0_. Then, all stretches defined by *x_i_*/*X_i_* depend only on *x*, the current configuration coordinate or equivalently on *X*, the coordinate in the reference configuration before loading. The hyperelastic energy, *W*, is a function of the principal stretches: *W*(*λ*_1_, *λ*_2_, *λ*_3_) with the Cauchy stress given by3.1
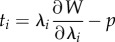


*λ_i_* means the principal stretch in the *i* direction, the ratio between the current length *l_i_* of the sample and the initial length *L_i_*, in the same direction. *λ_i_* (where the index *i* takes the value 1, 2, 3 meaning respectively *x*, *y*, *z*) and *p* are *x*-dependent. The pressure *p* is a Lagrange multiplier, which ensures the incompressibility of the sample, given by3.2
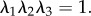


In addition, because of the mechanical equilibrium, we have3.3
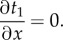


So, *t*_1_ is a constant along *x*. A similar equation applies for *t*_2_ and *t*_3_, which have also constant values in the sample. Applying the cancellation of the stress in the *Y-* and *Z*-direction, we obtain3.4
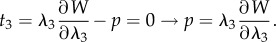


Equation (3.2) and (3.4) enable *λ*_2_ and *λ*_3_ to be solved as a function of *λ*_1_ to recover the Cauchy stress *t*_1_ as a function of only *λ*_1_. Our experiments determine the uniaxial force3.5
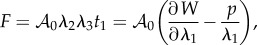
with 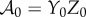. Note that *F* is directly proportional to the nominal stress [24].

The complete understanding of our experimental results requires a good model for the constitutive law of tissues with fibres but also a systematic study of these models versus the orientation, dispersion and failure. The experimental analysis, including the determination of fibre orientation and dispersion by using optical methods, will be considered in a future study. Here, we focused on the analysis of our results by using biomechanical models that incorporate fibre behaviour at a given orientation and dispersion. Because most of the continuum biomechanics models superpose the elasticity of the ground state to the one of fibres, we begin by the ground-state representation.

### 3.2. The low stretch biomechanics model

For the ground matrix and applied homogeneous strain, we apply two classical models, the Mooney–Rivlin model and the Valandis–Landel model [24]. The energy density for the Mooney–Rivlin model is3.6
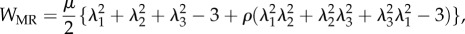
where the *λ_i_*, as before, means the principal stretch 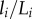. It is possible to replace *λ_i_* by the first invariants *I*_1_ and *I*_2_. The first invariant is 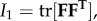 whereas the second *I*_2_ is defined by 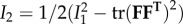 with the deformation gradient being the tensor: 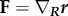. In equation (3.6), *μ* is called the infinitesimal shear modulus [24], having the dimension of pressure (Pa). The Mooney–Rivlin model is an expansion of the elastic energy density limited to *I*_1_ and *I*_2_. To ensure convexity at low strains, the dimensionless parameter *ρ* can be negative but larger than −1, a restriction that may be revisited for fibrous elasticity.

We have also considered the Valandis–Landel model, which allows a better representation of large rubber deformation, and the elastic energy density reads [24]3.7
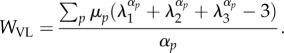


Both models recover the neo-Hookean model with *ρ* = 0 in the Mooney–Rivlin model and with *α_p_* = 2 in the Valandis–Landel model. Note that convexity of the elastic energy and ellipticity is also required to avoid irrelevant singularities in the material behaviour; these properties are difficult to prove in practice in three dimensions for arbitrary models and deformation which is why we restrict ourselves to standard representations of elastic energy. For the Mooney–Rivlin model, *ρ* can be negative but, for convexity remain larger than –1. In this case, we obtain force versus stretch given by3.8
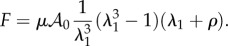


For low values of stretch (*λ*_1_ → 1)3.9
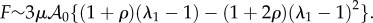


The uniaxial test results are compatible with the Mooney–Rivlin model, giving a negative initial curvature for *ρ* > −1/2, positive for −1 < *ρ* < −1/2. For the Valandis–Landel model, the result for *F* is not explicit but an expansion in series is possible. To quadratic order, it gives3.10
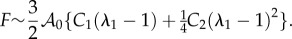
with
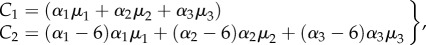
giving the possibility of having positive and negative initial curvatures as soon as one of the *α_i_*'s is between 0 and 6.

## 4. Fibre hyperelastic models

Fibres (e.g. collagen) are a consequence of the inflammatory process. They may be disordered, polydispersed, cross-linked with arbitrary orientations or have a well-defined orientation. They may also contain diverse bundle families. Under stretch, they can reorient themselves in the direction of the stretching or simply break as observed for the fabrics in reference [23]. We suspect that the network more or less stays in the tangent plane of the implant surface. It is also the *X*, *Y* plane of the samples from the surgical cut. However, we ignore their orientation in this plane. Biomechanical models exist for fibrous matter [25,26], but most of them concern the arteries and heart. There has not been a well-established model for fibrotic connective tissue. In addition, none of them seems to win unanimous support for arbitrary materials even if one of them is the most employed in theoretical biomechanics. Comparing our experimental data with models, we will make the simplest choice possible for the constitutive laws that we will use in our growth problem (see §5). We consider here the Gasser–Ogden–Holzapfel model [27] (called G–O–H model hereafter), and a more simple model presented first in reference [28], then extended in reference [29] for cross-linked fibres. Very few studies concern the stretch of cuboids, (except [30,31]) where the three-dimensions are fully involved. Peng and Cao experimentally examined the mechanics of woven composite fabrics both in experimental uniaxial tests and numerical simulations in two dimensions. Their experimental relationship between force and stretch is very similar to the present study with fibre breakage but indicates an ascending curvature at low stretch. Annaidh *et al.* [31] have performed a similar experiment for the dermis and propose a model for fully anisotropic three-dimensional collagen distribution with the G–O–H model [27]. Note that this model introduces only *I*_4_ and can be limited for strongly anisotropic materials where the invariant *I*_5_ has to be introduced [32,33]. The sensitivity of this coupling between invariants has been the subject of theoretical works such as [34]. Here, we take the viewpoint of the simplest model able to represent our data with the minimal set of independent parameters knowing that we are faced with fibre remodelling and breakage. First, we consider well-defined cross-linked oriented bundles, then we introduce dispersion and remodelling, and finally breakage.

### 4.1. An hyperelastic fibre model combined with Monney–Rivlin model at fixed orientation

Let us begin with the CB model [28] with fixed orientation *θ*_0_, *θ*_0_ being the angle between the fibres and the stretch direction (*X*-axis).4.1
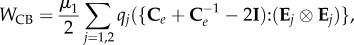
where *q_j_* is the material parameter indicating the fibre reinforcements along the direction **E***_j_*. For anisotropic materials and an in-plane cross-linked fibre networks, these parameters are a function of the density of fibres multiplied by the strength of these fibres in the *θ*_0_-direction. Assuming that the main directions are symmetric with respect to the *X*-axis with equal strength and density, we get4.2
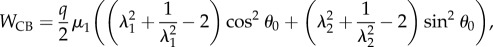
with anisotropy only determined by the orientation. This orientation can be dispersed and can remodel with stretching, inducing a decrease of *θ*_0_. This will be considered in §4. As a result of the lack of knowledge of a preferred orientation, we take 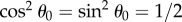, which also corresponds to an averaged fibre energy for a fully disorganized fibre network and we find in this case 4.3
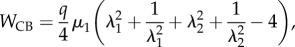
which simply modifies the coefficients of the Mooney–Rivlin contribution chosen for the ground matrix, making it stiffer. The elastic density energy is then the sum of both energies as 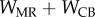. In this case, the calculus of the force can be achieved analytically and gives4.4
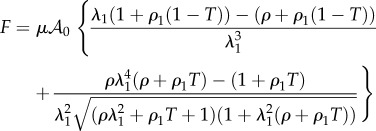
with 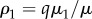, (*ρ*_1_ being a dimensionless number characterizing the strength of the fibre network), and 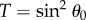 which gives to leading order4.5
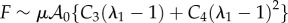
4.6
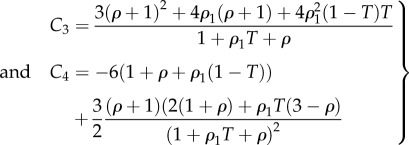


As a necessary but not sufficient condition, the model requires, *C*_3_ to be positive, because it is proportional to the shear modulus. Problems can occur when *ρ* is negative but only a negative *ρ* can allow a change in the sign of the curvature (force versus stretch). Because *C*_3_ vanishes for 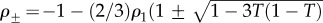, *ρ* can be negative and smaller than –1, the limit of stability of the Mooney–Rivlin model with fibres. Because we allow remodelling, the sign of *C*_3_ will also change with *T*, so we must work in a domain of parameters where *C*_3_ is always positive for any value of *T*, 0 ≤ *T* ≤ 1. However, another limitation comes from the fact that the force *F* can diverge. Indeed, looking at equation (4.4), a singularity automatically occurs as soon as 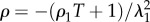, which occurs for negative *ρ* values, when we increase the stretching if the structure of the sample persists. Of course, this range of parameters must be eliminated if we keep this model.

### 4.2. Discussions on the parameters

**Figure 3.**
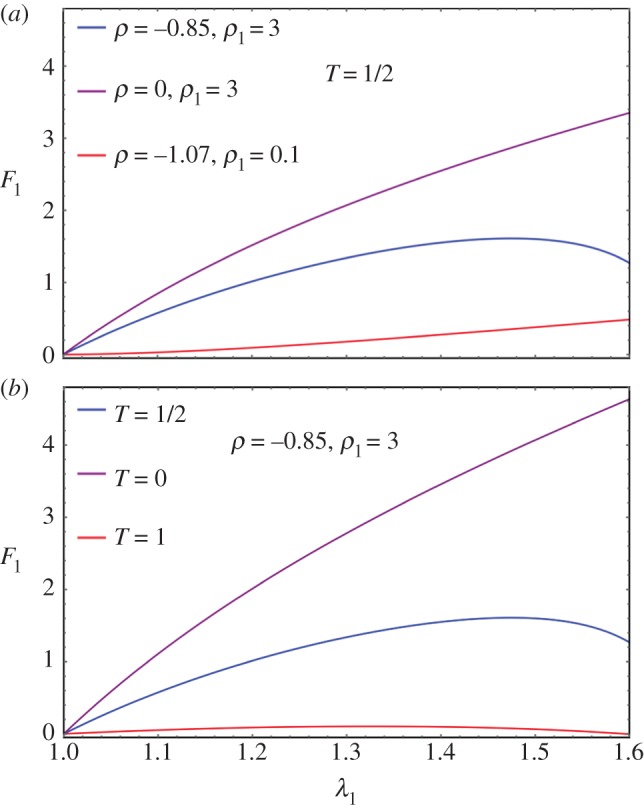
(*a*) Force versus stretch along the first direction for a sample treated by Mooney–Rivlin biomechanical energy and fully disordered fibres in the CB mode (*T* = 1/2). The shear modulus is chosen as unit, *ρ* and *ρ*_1_ varies in order to change the curvature at the origin according to equation (4.6). (*b*) Force versus stretch along the first direction for a sample treated with Mooney–Rivlin biomechanical energy and oriented fibres. The shear modulus is chosen as unit, *ρ* and *ρ*_1_ are fixed to give *a priori* a negative curvature with *ρ* = −0.85 and *ρ*_1_ = 3, but the fibre orientation 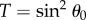 varies.

In §4.1, we select the Mooney–Rivlin model for the ground matrix, which explains the positive curvature of the curve force versus stretch, the classical model (neo-Hookean) being in favour of a negative curvature. If *ρ*_1_ = 0, one recovers *C*_3_ = 3(1 + *ρ*) and *C*_4_ = −3(1 + 2*ρ*) which seems to be adequate if *ρ* > −1. The initial curvature is positive for 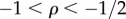 and negative above –1/2, which allows our data to be described with a single and simple model, changing only the coefficient *ρ*. Adding fibres increases the mechanical stability of the model. Various results are plotted in figure 3 for fully disoriented samples and for various orientations. In figure 3*a*, the force versus stretch exhibits appropriate behaviour: the force remains positive with increasing stretch and presents no singularity. In addition, it exhibits both positive and negative curvatures according to the *ρ*_1_ values. When 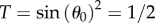, the possibility of having a positive curvature is reached close to the lower bound *ρ*_−_. Close to *ρ_+_*, the initial curvature is negative. Changing the orientation of fibres may give singularities of the force showing that the validity of the model depends tremendously on the coefficient. However, such behaviour occurs in all the constitutive laws of soft tissues. Figure 3*b* demonstrates the effect of fibre orientation. Indeed, these calculations achieved at fixed orientation cannot explain the effect of remodelling, because it is expected that the fibres orient themselves in the direction of the stretch as it increases. In the following, we examine fibre dispersion, the remodelling that occurs during the experiment.

### 4.3. Fibre dispersion and remodelling

In the previous paragraph, the bundles of fibres are assumed strictly oriented, symmetric to the stretched direction. For simplicity, we consider an in-plane distribution of fibres 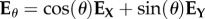 dispersed around the orientation 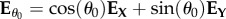 varying between 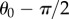 and 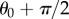, with the normalization condition4.7
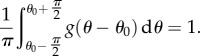


For simplicity and the uncertainty of the mean orientation *θ*_0_, it is assumed that *θ*_0_ = 0, and4.8
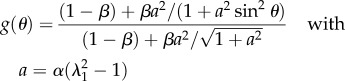


describing in-plane fibre distribution that is symmetric to the stretched direction, with one part that remains fully disorganized 1 − *β* and a part, *β* that reorients in the direction of the stretch as the experiment proceeds with increasing stretching *λ*_1_ > 1. The coefficient *a* is responsible for the remodelling process, often considered as being a very slow process, *α* being an efficiency parameter. The two-dimensional structure tensor [27,31,34] is then given as follows4.9
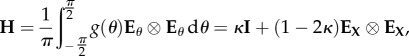
where4.10
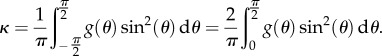


**Figure 4.**
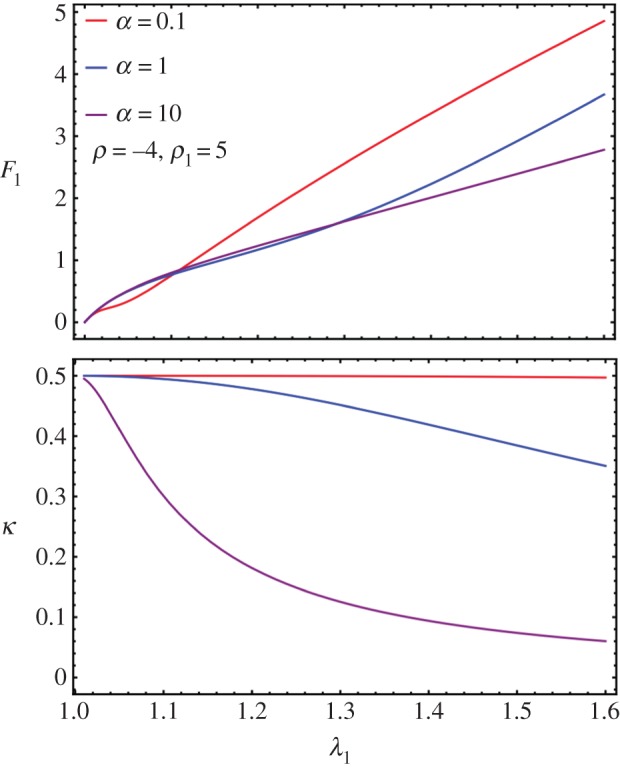
(*a*) Force versus stretch in a case of remodelling for values of the *α* parameters. (*b*) Modification of the fibre orientation *κ* as a function of the stretch *λ*_1_ in a case of remodelling according to different values of *α*.

Once the calculation of the structure tensor is performed, one gets a diagonal tensor: 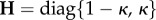, the *κ* value having the same significance as 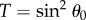 in the strictly oriented case, but evolving with 
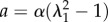
 owing to remodelling (reorientation) with the stretch. In figure 4, we give an example of *κ* choosing *β* = 1, *α* = 0.1, 1, 10 showing the decrease of *κ* from 1/2 to 0 as the stretching proceeds. Corresponding results for force versus stretch are also given in figure 4 to compare with the experimental results in figure 2. However, the results disagree with the experiments, so fibre remodelling decreases the sensitivity of the force to large stretch values.

### 4.4. Breakage of fibres

Fibre breakage can be observed on our experimental curves as small discontinuous jumps, which do not excessively distort the global tendency. Here, we aim to extend the fibre model to breakage. Assuming that there is no breakage before stretching, we will take **H** as the initial condition. A fibre, oriented along **E***_*θ*_* breaks if the stretch along this direction is above a typical toughness value given by 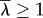, the same for each fibre such that breakage occurs if4.11
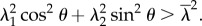


For stretching 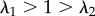, equation (4.11) is equivalent to4.12
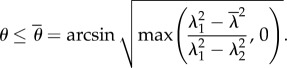


Breakage occurs only if 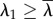. So the structural tensor **H** depends on *λ*_1_, *λ*_2_ and 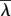 via 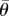 by the following modified formula4.13
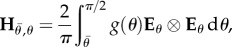
which can be simplified to4.14
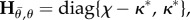
where4.15
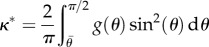
and4.16
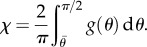


Note that κ* = *κ* and *χ* = 1 when 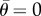, thus 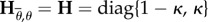 where we recover the remodelling case.

*χ* actually provides the overall strength of distributed fibres left from breaking. When *β* = 1, by equation (4.8),4.17
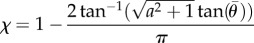
4.18
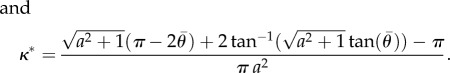


Note that when *a* = 0 (no remodelling), we have 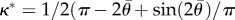. When 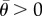 and *a* → ∞, we have *χ* → 0 and *κ* → 0. When 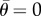, we have *χ* = 1, regardless of *a*. The corresponding modified CB model considering dispersion and breakage is transformed into4.19
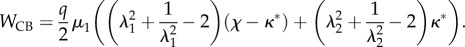


**Figure 5.**
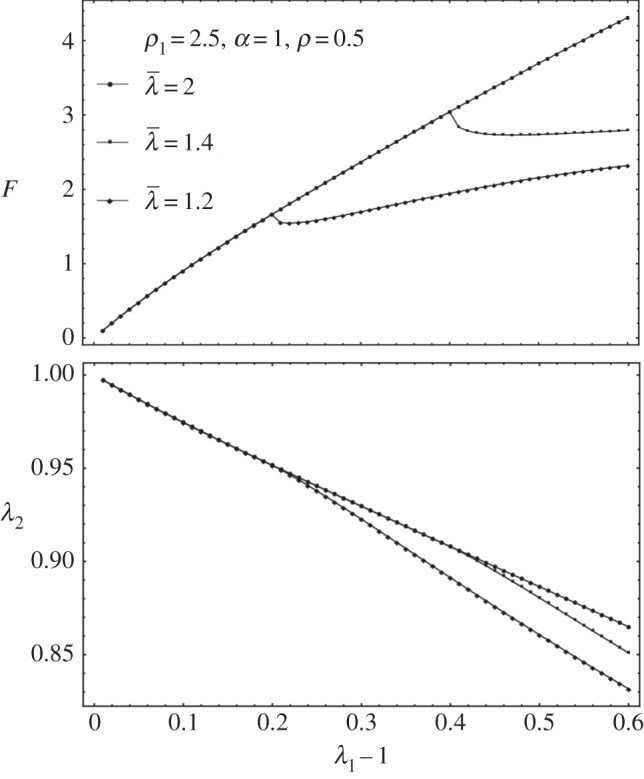
Fibre breakage: (*a*) *F* drops and increases slightly during further stretches. (*b*) Lateral stretch *λ*_2_ versus longitudinal stretch *λ*_1_ when fibre breakage occurs.

For 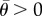, we can no longer solve *λ*_2_ by cancelling *t*_2_ explicitly. So we numerically solve both *λ*_2_ and *F* together as a function of *λ*_1_. When there is no breakage, the numerical solver in Mathematica is consistent with the analytical formula in equation (4.4). See figure 5 for the existence of a sudden drop in *F* followed either by a quasi-plateau or an ascending curve according to the different values of the breaking threshold 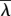. It seems the remodelling parameter *α* does not control the trend of *F* after breaking, and it is *ρ*_1_ and *ρ* that mostly controls the trend. So it is the ratio between the fibre stiffness and the ground material that controls *F* after breaking.

### 4.5. Exploration of the G–O–H model

From reference [31], we can see that the G–O–H model, considering distributional fibres with the neo-Hookean ground material, can reproduce the upward concavity of the stress–strain curve. Although concavities of the stress–strain and force–strain are not equivalent, we try to explore what the G–O–H model can provide for our case. For multiple families of fibres, we have4.20
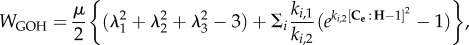
where *k_i,j_*'s are positive dimensionless stiffness parameters. *k_i,_*_1_ is related to the stiffness of the fibres in the small strain regime, whereas *k_i,_*_2_ is related to large strain-stiffening behaviour of the fibres [31]. This suggests that if the breaking is to be considered at the yielding phase, it should only modify *k_i,_*_2_, not *k_i,_*_1_. If we consider two families of fibres symmetric to the stretched direction with the angle deviation ±*θ*_0_, as in reference [31], we have4.21
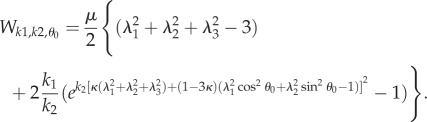


**Figure 6.**
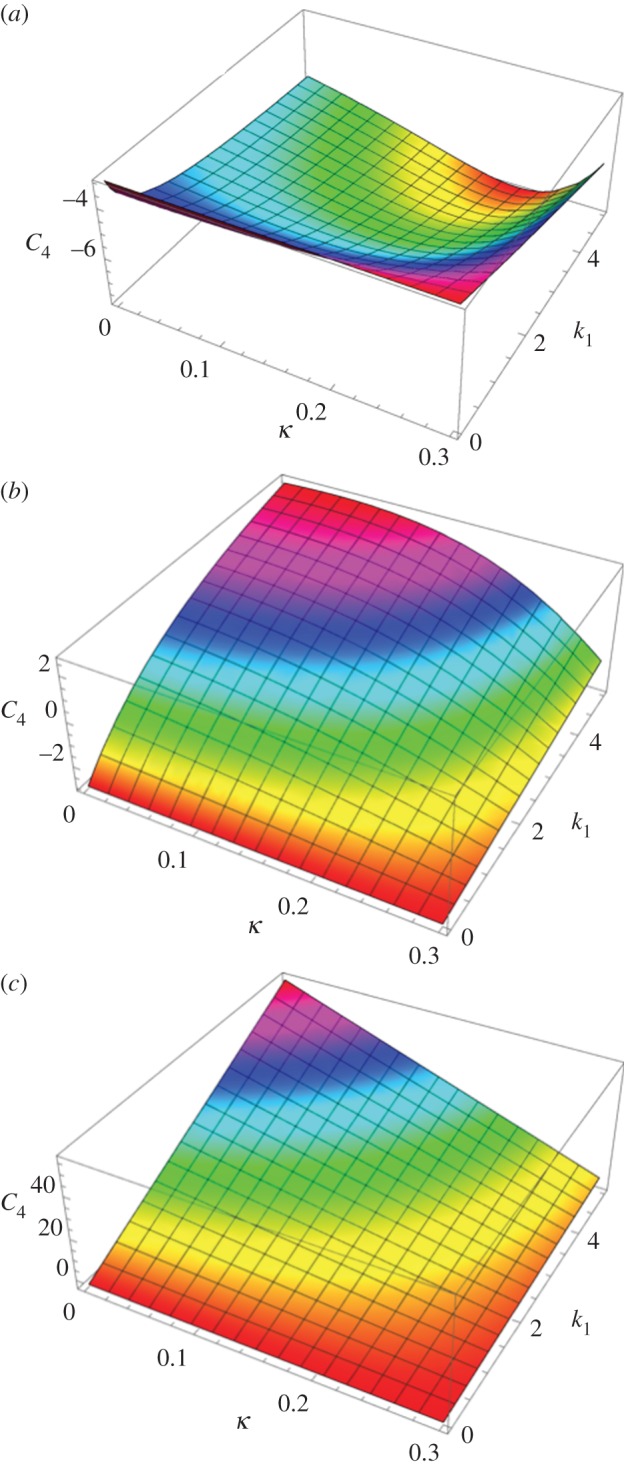
The initial curvature coefficient *C*_4_ deduced from the G–O–H model, for orientation of the fibre perpendicular to the stretching direction (*θ*_0_ = *π*/2) (*a*), randomly distributed *θ*_0_ = *π*/4 (*b*) and parallel to the force *θ*_0_ = 0 (*c*) as a function of *k*_1_ and of the dispersion coefficient *κ* (according to the definition given in equation (4.21)). Note the extreme sensitivity of this coefficient to *k*_1_, which is equal to *C*_4_ = −3 for *k*_1_ = 0 (neo-Hookean value) and reaches *C*_4_ = 45 for *k*_1_ = 4, whereas for high levels of dispersion, which coincides with *κ* = 1/3, we get *C*_4_ = −3.

The *κ* coefficient gives the level of dispersion around the direction fixed by *θ*_0_. First, let us see what the model does without breakage depending on *k*_1_ and *k*_2_. Let us calculate the low stretch behaviour, because it may be very useful as an indication of the parameter range for fitting. We consider the three typical orientations, distributed along the stretched direction *θ*_0_ = *π*/2, *θ*_0_ = ±*π*/4 and along the transverse direction *θ*_0_ = 0 given different level of dispersion *κ*. We then calculate the initial stiffness *C*_3_ given with or without dispersion and the initial curvature *C*_4_ (given here for *κ* = 0 for simplicity but plotted in figure 6), according to equation (4.5). For *θ*_0_ = *π*/2, we have4.22
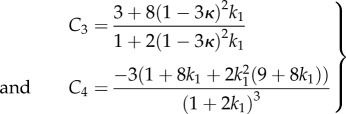

Note that for this orientation, *C*_4_, is always negative. For *θ*_0_ = *π*/4, we have4.23
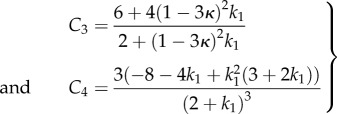


Note that for *θ*_0_ = *π*/4 the initial curvature of the force versus stretch changes for *k*_1_ > 1.5265 from negative to positive. For *θ*_0_ = 0 (orientation of the fibres along the forcing), we have4.24
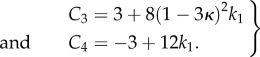


In this case, *C*_4_ changes sign from negative to positive for *k*_1_ > 0.25. The effect of the dispersion is shown in figure 6, which also demonstrates the extreme effectiveness of the dispersion coefficient *κ* on the *C*_4_ value. When the fibre orientation is unknown, extracting any conclusion is difficult from experimental tests. We also have tested the two-dimensional G–O–H model in detail for fully in-plane fibre distribution. Interestingly, this model gives the same coefficients *C*_3_ and *C*_4_ as the three-dimensional model when *κ* = 0.

**Figure 7.**
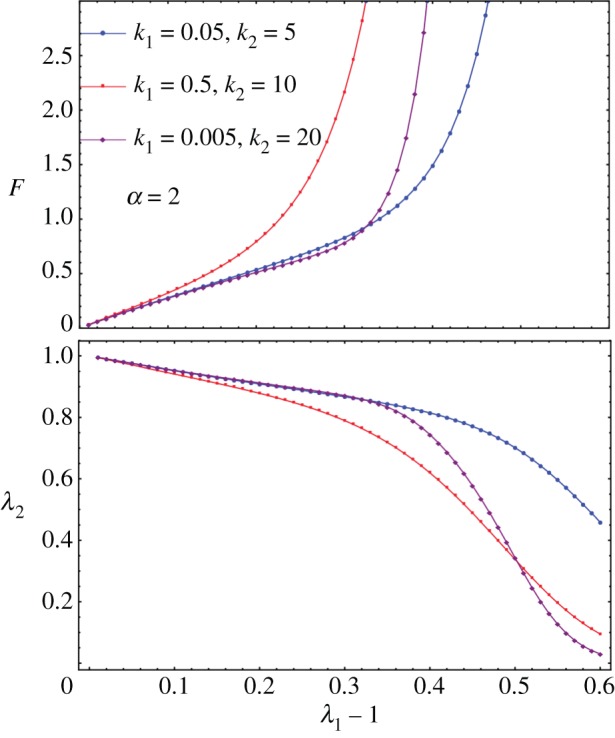
*F* versus *λ*_1_ for diversities of *k*_1_ and *k*_2_ when *α* = 2. For larger *α*, the blow-up is more obvious, which is not shown here.

**Figure 8.**
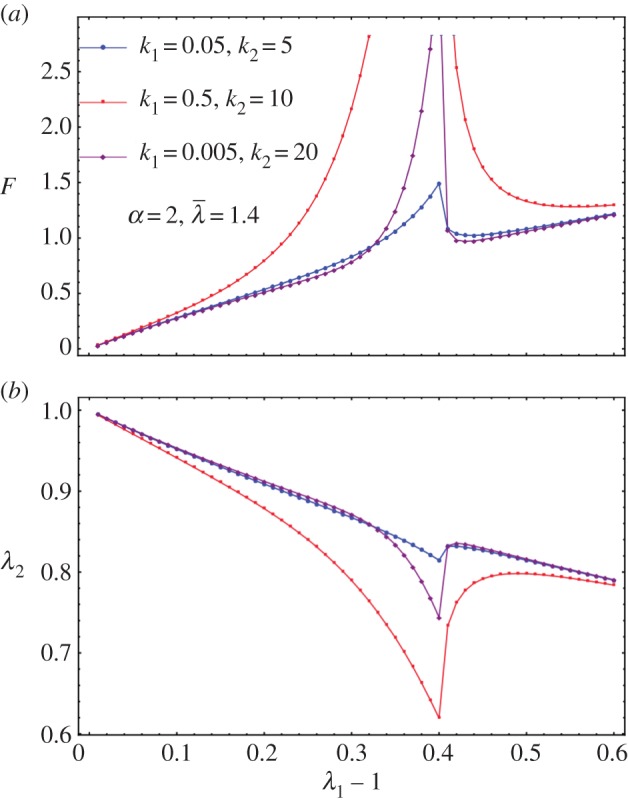
(*a*) *F* versus *λ*_1_ for diversities of *k*_1_ and *k*_2_ when *α* = 2 and 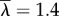 as an example. (*b*) *λ*_2_ versus *λ*_1_ − 1 for diversities of *k*_1_ and *k*_2_ when *α* = 2 and 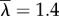 as an example.

We can easily apply fibre remodelling and breaking on this model by considering the large strain stiffness parameter *k*_2_ affected by *χ* (from equation (4.16)). The overall strength of distributed fibres after breakage reads4.25
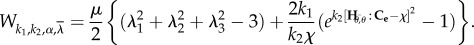
Now, we have the remodelling parameter *α* (the same as in equation (4.8)) and the breaking threshold 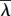 (the same as in equation (4.11)) and we explore the effect of them on top of *k*_1_ and *k*_2_. The remodelling of fibres (figure 7) contributes to the increased sensitivity of the force to the stretch; however, the breaking is probably more interesting and has to be compared with the Mooney–Rivlin–CB fibre model. In figure 8, we show the effect of breaking on the force versus stretch in parallel to *λ*_2_ versus the stretch *λ*_1_. It is similar to applying the Mooney–Rivlin–CB fibre model. One of the interesting things is we may be able to identify fibre breakage from remodelling in the experiment. Remodelling contributes to more compression in *λ*_2_ (shown in figure 8); however, breakage contributes to an instant increase of *λ*_2_ after breaking. This may be caught during the experiment (figure 2).

### 4.6. Comparison of the models for parameter determination of the hyperelastic laws

**Figure 9.**
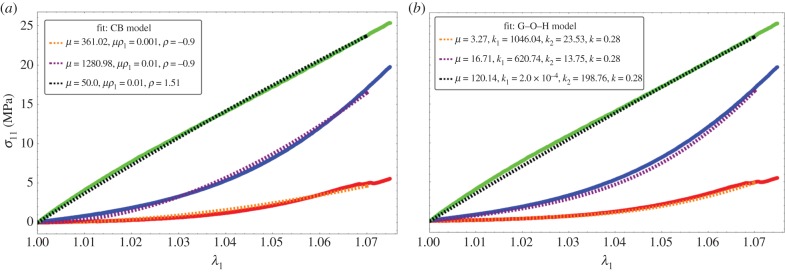
Fitting values of the nominal stress in the CB model (*a*) and three-dimensional G–O–H model (*b*) corresponding to the experimental curves of figure 2*a*. Coloured lines are experimental results, black dotted lines correspond to theoretical model with the best-fit parameters. Note the strong nonlinearities of the tissue at low stretch values.

**Figure 10.**
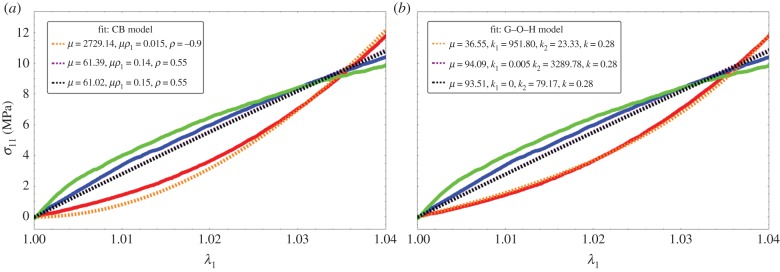
Fitting values of the nominal stress in the CB model (*a*) and three-dimensional G–O–H model (*b*) corresponding to the experimental curves of figure 2*b*. Coloured lines are experimental results, black dotted lines correspond to the theoretical model with the best-fit parameters. Note the strong nonlinearities at low stretch values.

In the previous theoretical study, we compared two different fibre models and their ability to recover traces of our force–stretch curve. Our main limitations are the finite size of our surgical tissues and the lack of information of the orientations inside the sample. Focusing on the simplest model, CB, the fit obtained in figures 9*a* and 10*a* is satisfactory compared with the most established three-dimensional G–O–H model, which has added parameters such as the dispersion coefficient *κ*. According to equation (4.6) and figure 3, the CB model gives a positive curvature at low stretch for *ρ* < −0.5 and *ρ*_1_ ∼ 0, which may correspond to a capsule of low Baker grade with low fibre density. When the fibre density increases with a well-defined organization, as for contracted capsules [19], *ρ*_1_ increases and the curvature becomes negative. This model explains the main characteristics of our biomechanical experiments well (figure 2*a,b*) with a change of parameters, which physically corresponds to the histological findings of Bui *et al.* [19]. In addition, we explored the two-dimensional version of the G–O–H model, which is justified by the small thickness of the capsular tissue; however, in this case, the fit was unsatisfactory. Our theoretical analysis, however, proves the extreme sensitivity of the parameters, especially for G–O–H, and the fact that the orientation is unknown produces serious uncertainty regarding the determination of the constitutive law of these tissues. For the fit, we always take the orientation at *θ*_0_ = *π*/4. The real advantage of the CB model is to provide an analytical answer, and that remodelling and fibre breakdown can be incorporated easily. To discriminate between the models, it would be useful to have experimental information regarding the transverse direction *y* because the models behave differently at breakdown. Both models show different lateral contraction *λ*_2_ (compare figure 5 and figure 8), which may be experimentally verified in future studies. For the CB model, unphysical singularity occurs for large stretching for a set of parameters that are well behaved at low stretch. However, at large stretches, the sample structure may evolve via plastic deformation. These results, to the authors' knowledge, however, are the first concerning fibrotic tissue in human beings, and are enough to explain the problems induced by an inflammatory reaction to implants. In §4.5, we discuss the consequences of the growth of this capsule.

## 5. Evaluation of the stresses during capsule formation

**Figure 11.**
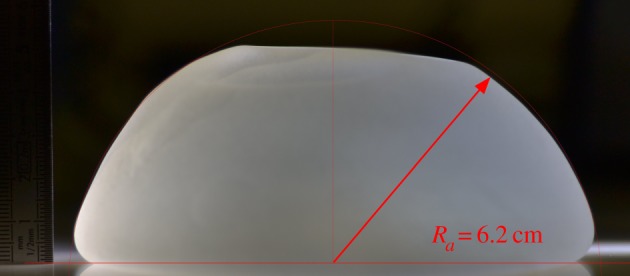
Typical implant for evaluation of the Young modulus using compression and the elastic Hertz contact theory.

**Figure 12.**
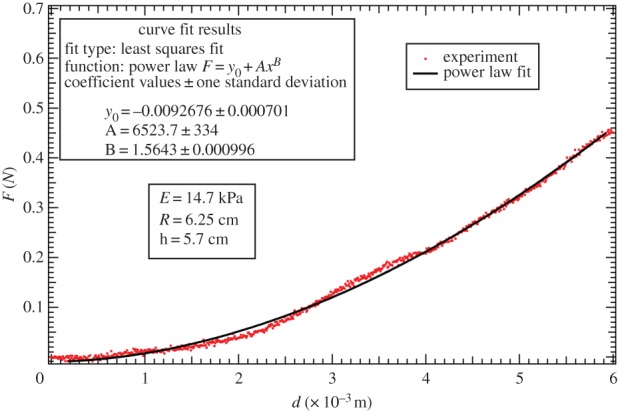
Data fit of the force versus the compression displacement using the linear elastic Hertz contact theory for the deformation of the implant. Power law fit: 
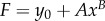
. Estimation of the Young modulus, derived from the method of Hertz contact [43], is about 14.7 kPa.

The capsule is the result of the growth of a thin layer of connective tissue. In case of breast reconstruction, the fat and gland tissues are eliminated, and the implant is covered simply by the nascent capsule and skin. The capsule adheres to the implant, having no possibility to slide or detach. As a result, the growth process is mainly directed along the implant along the normal, making the growth anisotropic and generating automatically compressive stresses. These stresses, which appear in the tissue and the implant, are called passive. They exist each time the growth is forced in a direction owing to the implant and consist of a particular case of Biot instability [35]. They also exist for an ordinary swelling process of polymeric gel attached on a solid substrate as shown by Tanaka [36] in pioneering works and, among others, [37–41] (for a review see reference [42]). In addition, these stresses induced a buckling of the growing layer that distorts the implant. As mentioned, the implant has a semi-spherical geometry with a radius of *R_a_* = 6.2 cm. The spherical geometry protects the implant from stresses; however, as soon as the sphericity is lost, deformation and stresses occur also in the implant. For completeness, we evaluate the Young modulus of our silicone implant by the method of Hertz contact [43] (figure 11) and obtain 14.7 kPa. Although the theory concerns Hookean elasticity, and silicone is mostly neo-Hookean, we get a good fit, as shown by figure 12. Our stress estimation needs to be compared with shear moduli for healthy breast fat, which varies with the experimental technique between 2 kPa [44] up to 20 kPa [45] and stiffens in the vicinity of lesions: 45.6 kPa for benign one up to 146 kPa for malignant [46]. These results have been derived with elasto-sonography *in vivo*. We aim now to evaluate the stresses in the capsular tissue and perhaps to evaluate the active stresses if any. Let us evaluate first the radial deformation owing to growth.

### 5.1. The geometric and elastic deformation tensor

**Figure 13.**
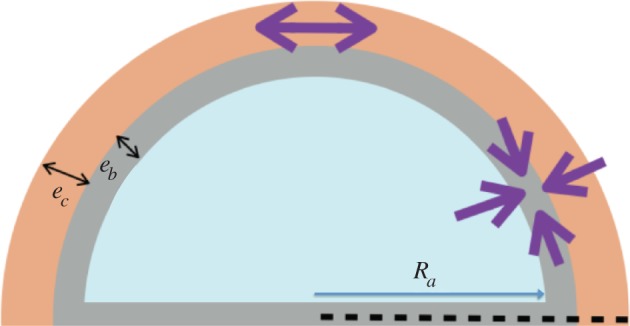
Schematic of the implant, the capsule and the skin layer.

We consider a three-layered system with spherical symmetry (figure 13). The implant having the shape of a cap of radius *R_a_*, the capsule occupies the space between *R_a_* and *R_b_* and the thin skin layer the space between *R_b_* and *R_c_*. Because of the presence of the fibres, the growth is assumed anisotropic and the growth tensor is 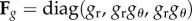. *g*_r_ represents the relative growth in the radial direction, whereas *g_*θ*_* is an anisotropic coefficient, identical along meridians and parallels for simplification. The relative volume increase *J_G_* is then given by 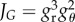. In addition, the tissue can be prestretched because of active cells and these stretches appear parallel to the implant surface. The prestretch tensor is also a compressive one, 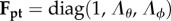, so the deformation gradient becomes 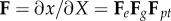 and the elastic tensor is then5.1
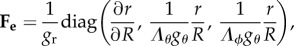

Note that both 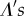 are smaller than 1 in the case of a compressive active stretch, which is expected here, as a spontaneous reaction of the immune system against the implant. The local volume increase, *J_G_*, is larger than 1 when the capsule grows. Owing to the hypothesis of incompressibility, which is valid for living tissues and elastomers, 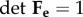, so5.2
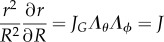


with *J* = 1 for the implant and also the skin. Focusing only on the determination of the order of magnitude for the stresses involved in the capsule formation, we restrict to a base state of deformation, which respects the spherical geometry, and we do not treat the full buckling of the capsule with the implant. *J* represents a control parameter of the buckling process, being a growing function of time if the pathology persists. This full buckling instability is rather technical, some examples can be found in reference [15] for the anisotropic spherical case and in references [18,34] for the cylindrical geometry. The evaluation of the threshold instability given by the critical radial geometric stretch *r*/*R* is derived via the solution of an eigenvalue problem involving the control parameters which are, here, the prestretch values and the growth eigenvalues *g*_r_ and *g*_*θ*_. Restricting to the simplest radial solution with an undeformed implant, with the capsule expanding radially, we get the new position of the layers which is5.3
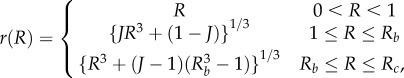
where *r*(*R*) is the new position of the layer, which was initially at a radius *R*. *r*(*R*) respects the continuity at the border zones. Each layer is very thin. In equation (5.3) and in the following, the radius of the implant is chosen as the length unit *R*_*a*_.

### 5.2. Radial stresses

For simplicity, let us assume 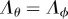. In the spherical coordinate system and in the current configuration, the equilibrium equation for the Cauchy stress 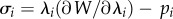 (*i* refers to each layer) gives5.4
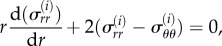
which we can transform using the elastic stretch 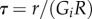 into5.5
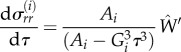
with *A_i_*, the coefficient of *R*^3^ into equation (5.3): *A_i_* = *J* for the capsule layer and *A_i_* = 1 both in the implant and the skin. 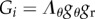 and *A_i_* = *J* for the capsule layer while *G_i_* = 1 for the implant and the skin. As in references [15,24], 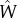 is the elastic energy density for incompressible material 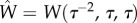, function of a unique stretch eigenvalue, 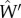 being its derivative with respect to the stretch *τ*. This simplification assumes transversely isotropy. In practice, the radius of the implant (*R_a_* = 6.2 cm) is larger than the thickness of the layers, of the order of millimetres, both for the capsule and the dermis. So the relative thickness *e_b_* of the capsule and *e_c_* the dermis are small dimensionless parameters giving 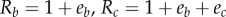 in the reference configuration. To fix the stresses, we begin with the skin and we impose at the outer surface of radius, 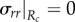, corresponding to mechanical equilibrium. So the radial stress inside the skin layer (where no distinction is made between epidermis and dermis) is then given by5.6
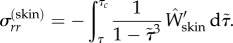


Remember that 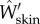 is scaled by 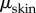 the shear modulus coefficient of the skin density energy and *τ*_skin_ is given by5.7
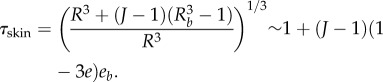
The skin is obviously stretched; however, the stretch differs from 1 inside the skin by a second-order coefficient, because *e* is the distance from *R_b_*. So the compression of the skin is given by5.8
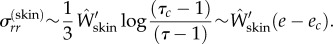
Because *e* is a dimensionless number corresponding to the radial position *R* – *R_b_* divided by the implant radius *R_a_*, we recover the Laplace Law where the surface tension *γ* can be identified as 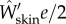 with *e* given now in international units. For the growing capsule, we have5.9
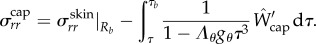


However, owing to the small thickness of this capsule compared with the initial radius of curvature of the implant, we can estimate the elastic stretch inside the capsule to be5.10
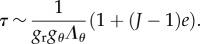


In this limit of small thickness, *τ* is close to a constant inside the capsule, being given by 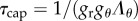. It is to be noted that in practice it is difficult to estimate these factors independently and only 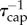 can be estimated. The radial stress is then5.11
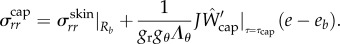


The capsule remains rather thin except for very advanced cases, with a thickness that varies from half a millimetre (corresponding to Baker grade I) to 2 or 3 mm (for Baker grade III), which remains small compared with a radius of curvature of order 6 cm, for the implant. Again, the radial stress scales as the thickness of the layers. Finally, in the implant, a radial stress exists at the border given by 
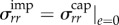
, but our radially symmetric solution does not treat implant deformation. Nevertheless, for a layer size that doubles from grade I to grade III, a buckling of the layers occurs as shown in [12,15], the hoop stress being compressive. So now, we evaluate this quantity in each layer.

### 5.3. Layer-by-layer evaluation of the order of magnitude for radial and hoop stresses

In each layer, in radial geometry, we have5.12
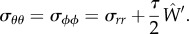


For the skin, taking into account equations ((5.7) and (5.8)), we obtain5.13
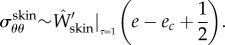


This calculation assumes that there is no proliferation of dermal skin induced by this tensile state, which is probably not true. This point will be discussed later. In the capsular tissue, we have5.14
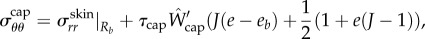
with 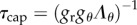 and 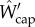 being defined for *τ* = *τ*_cap_ value, which is smaller than 1. So the stress is compressive. It is important to note that the hoop stress is quite independent of the relative thickness of the layer.

### 5.4. Stresses, buckling and pain

Here, we aim to estimate the mechanical stresses during fibrosis. Between a capsule of size 0.5 mm, a size we take as the initial condition, to a capsule of 2 mm, the size is multiplied by 4, which is above the stability limit of a spherical layer according to [12,15]. Indeed, the threshold for buckling instability is of the order of 1.5 for *g*_r_ for a stiff substrate and decreases when the substrate is much softer than the layer. We can surely claim that in grade III, we are above the stability of the spherical symmetry and the whole system will buckle. In addition, our mechanical tests indicate that the tissue itself becomes more and more stiff, the stiffness being confirmed clinically, as part of the diagnosis. Nevertheless, it is interesting to evaluate the stresses involved, which manifest themselves by deforming the breast and implant, explaining the pain, and occasionally, the rupture of the implant. Let us begin with the skin. There are few data on the skin, including the dermis, *in vivo*. Here again, the thickness involved is of the order of millimetres. The Langer lines [31], which give the main orientation of the collagen inside the dermis of the skin, can be assumed along parallels and meridians at the level of the breast. Although we know that the skin's elasticity varies a lot along the body, we are not aware of a study of the skin at the level of the breast, for young women. In reference [31], a very precise analysis comprising biopsies, traction measurements and modelling was performed, confirming that the Langer lines of surgeons correspond to the main orientation of the collagen fibres of the dermis and the G–O–H model was shown to correctly represent the skin's elasticity in a range of traction identical to our traction test. The structural parameters were evaluated with the coefficients *μ*_skin_ = 0.2014 MPa, *k*_1_ = 243.6 and *k*_2_ = 0.1327, whereas the dispersion coefficient *κ* = 0.1404. The skin is stretched by the growth of the capsule. Keeping the G–O–H model of the skin [31], we find 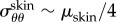 so the tension is 50 kPa. This number can be overestimated, however, because it assumes no cell proliferation. Being under tension, so under the homeostatic threshold, skin can grow to relax this tensile effect. In the capsular tissue, *τ* is a quantity that is significantly smaller than 1 and we will choose 1/2 as an appropriate estimation. Taking this value, we derived 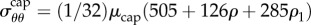. In the examined cases, we obtain a compressive stress varying between 7 and 10 MPa. This estimation is several orders of magnitude higher than the values of breast fat and may explain the sensation of stiffness of the capsule, the discomfort and the pain induced by nerve compression.

## 6. Conclusion

Here, we present a biomechanical study of the contracture of the breast capsule at different degrees of fibrosis. A tensile test experiment of thin samples obtained a few hours post-surgery enables the detection of two different constitutive laws that comply with the clinical classification. Baker grade I samples seems to present more anisotropy owing to well-oriented fibres, with breakage of the weakest filaments as the stretch increases. Baker grade III samples are stiffer; however, the orientation effect seems to be lost, indicating perhaps an increase in the internal disorder. The experiment covers stretch values of 1.6 corresponding to an elongation of 60%. We test two models of cuboid fibrotic tissues under tension with the difficulty that these models, although common in the literature of biomechanics, exhibit singular behaviour at large strains for part of the range of the parameters which is difficult to predict *a priori*. However, an estimate of the parameters corresponding to our results is possible, allowing estimation of the stresses. Our conclusion is that the contracted tissue is stiffer in the normal capsule (grade I) and severe capsule (III/IV) compared with the stiffness of the implant and the fat of the breast. This explains the discomfort if the fibrotic tissue grows as a result of the inflammatory reaction. In addition, a buckling instability is expected, beginning at grade III, leading to painful distortion of the implant (grade IV), unaesthetic appearance and sometimes implant rupture. This is also explained by the ratio between the shear modulus of the implant and the contractile hoop stress of 3%. The existence of active cells (myofibroblasts) can also be suspected at grade III/IV. However, several tests done on the samples with arbitrary cuts as performed in references [5,6] do not reveal the existence of pre-stresses. Pre-stretches are automatically included in the model, whereas pre-stresses can be introduced following the analysis of reference [17]. The study of fibrous tissues as a consequence of the immune system and inflammatory response is not limited to capsular contracture and may be applied to other disease entities such as cancer and severe obesity for example. Future work will concern more advanced critical contractors and the relation between the structure at the microscopic scale and the constitutive elastic laws valid at macroscopic scales.
